# Microvascular and Morphologic Changes of the Macula over Lifetime

**DOI:** 10.3390/life12040568

**Published:** 2022-04-11

**Authors:** Mael Lever, Ying Chen, Moritz Glaser, Jan Darius Unterlauft, Claudia Lommatzsch, Nikolaos E. Bechrakis, Michael R. R. Böhm

**Affiliations:** 1 Department of Ophthalmology, University Hospital Essen, 45147 Essen, Germany; ying.chen@uk-essen.de (Y.C.); nikolaos.bechrakis@uk-essen.de (N.E.B.); michael.boehm@uni-due.de (M.R.R.B.); 2 Achim Wessing Institute for Imaging in Ophthalmology, University Hospital Essen, 45147 Essen, Germany; moritz.glaser89@gmail.com (M.G.); claudia.lommatzsch@augenfranziskus.de (C.L.); 3 Department of Ophthalmology, University Hospital of Ophthalmology, Inselspital, 3010 Bern, Switzerland; jandarius.unterlauft@insel.ch; 4 Department of Ophthalmology, St. Franziskus-Hospital Münster, 48145 Münster, Germany; 5 Department of Ophthalmology, University of Lübeck, 23552 Lübeck, Germany

**Keywords:** macula, macular segmentation, aging, optical coherence tomography-angiography, vascular disease, capillary density, blood flow

## Abstract

In ocular, neurologic, and cardiovascular diseases, macular segmentation data from spectral-domain optical coherence tomography (SD-OCT) provide morphologic, and OCT-angiography (OCTA) results give microvascular information about the macula. Age was shown to influence both methods’ measurements. To further characterize this association, macular SD-OCT and OCTA changes were investigated in a population of juvenile, adult, and older individuals. Macular segment thickness and superficial (SCP) and deep plexus (DCP) vascular density (VD) of 157 healthy individuals aged 10–79 years were analyzed retrospectively. One-way analysis of variance (ANOVA) was used to compare age groups. The association between macular segmentation and OCTA parameters and between these and age was evaluated using linear regression. ANOVA and linear regression analysis showed a thickness decrease in the whole macular and in the ganglion cell and inner plexiform layers with age. While the foveal avascular zone area remained constant between age groups, VD of the SCP and DCP also decreased with age. In multiple linear regression, SCP and DCP VD were associated with inner macular segment thickness in an age-independent way. To conclude, the age-related microvascular and morphological changes in the macula described in this study can contribute to improving the understanding of macular aging processes and better interpreting OCT(A) results in healthy individuals and patients suffering from various retinal diseases.

## 1. Introduction

Over a lifetime, the eye is subject to functional alterations such as a reduction of visual acuity, of contrast and motion sensitivity, which are attributable to morphological rather than optical changes. Histological studies have revealed a thinning of the retina and, more interestingly, of the macula with age [1]. Technical advancements of the past decades led to optical coherence tomography (OCT), which now allows the precise measurements of these structural changes in vivo.

OCT provides a fast, contactless, highly reproducible [2,3], objective, and 3-dimensional visualization of the retina and of the optic nerve head [4]. For this, the light emitted from a diode or laser, which is reflected from the retinal layers, is compared to light reflected from a reference mirror by interferometry of low coherence. Spectral-domain OCT (SD-OCT) represents an important evolution of the original method, providing an increased axial resolution, thus allowing for computer-assisted retinal layer segmentation to enhance the quality and precision of the information gained about the retinal structure [5]. OCT studies have reproduced the changes observed histologically within the macula [6,7], and OCT has become a crucial diagnostic method for certain ophthalmic conditions. In particular, SD-OCT macular segmentation is now utilized in the management of age-related macular degeneration [8] and glaucoma [9,10,11] and was shown capable of detecting disease-specific alterations in other neurological, vascular, and metabolic disorders such as multiple sclerosis [12], Parkinson’s [13] and Alzheimer’s diseases [14], and diabetes mellitus [15].

OCT-angiography (OCTA) is the result of further refinements of the original OCT-method. OCTA uses up to 100,000 A-scans per second to identify blood vessels by detecting moving elements like erythrocytes within retinal capillaries [16]. This data can be further analyzed to quantify retinal and choroidal blood vessel density (VD) without using an intravenous dye, as in fluoresceine angiography. In practice, the optic nerve head and macula can both be analyzed with high reproducibility [17]. At the macula, the VD of the superficial capillary plexus (SCP) in the ganglion cell layer and of the deep capillary plexus (DCP) located between the inner plexiform and outer nuclear layer were shown to be of clinical value, as well as the foveal avascular zone (FAZ) area [16]. Like macular segmentation, OCTA parameters seem to physiologically evolve with increasing age [18,19]. While OCTA does not belong to the clinical routine yet, its ability to characterize pathologies of the eye are multiple: the FAZ area was shown to correlate with visual acuity in patients with central vein occlusion [20], and OCTA can identify preclinical diabetic retinopathy [21]; OCTA can help diagnose and characterize glaucoma [11,22,23]; characteristic changes are also visible after non-arteritic anterior ischemic optic neuropathy [24]; in children, OCTA can identify microvascular alterations of eyes with a retinopathy of prematurity [25,26].

Macular segmentation using both SD-OCT and OCTA parameters was shown to be influenced by age. Awareness of this association is crucial to the interpretation of these measurements, since only changes beyond normal aging effects can be interpreted as pathologic. In addition, the fact that the vascular plexuses analyzed by OCTA are located within the macular segments, which are mainly affected by aging, is of great relevance since the interaction of vascular density and inner retinal thickness as diagnostic parameters is still insufficiently understood [11,23].

The aim of this study was to provide additional normal data about the structural (macular segmentation) and microvascular (capillary density) characteristics of the macula from childhood to older age, and to further study the correlation between these measurements.

## 2. Materials and Methods

### 2.1. Study Population and Design

All patients aged 10 to 89 years without any retinal disease or optic nerve pathology who received a macular OCT and OCTA between May and September 2019 at the Department of Ophthalmology of the University Hospital Essen, Germany, were analyzed retrospectively. Exclusion criteria for this study were a history of ocular trauma or intraocular surgery (except uncomplicated cataract surgery), refractive errors > 3 diopters, a pronounced lens opacity/cataract, the presence of relevant systemic diseases (e.g., cardiovascular or neurologic), current treatments with vasoactive medications. Patients with missing data of best corrected visual acuity (BCVA), intraocular pressure (IOP), anterior segment examination, and/or fundoscopy were also excluded from the study. Data of the eye with the highest OCT image quality were kept for further analysis. This study was conducted in accordance with the 1964 Declaration of Helsinki and was approved by the ethics committee of the University Hospital Essen, Germany (approval number: 19-8820-BO).

### 2.2. Data Acquisition

All patients were examined comprehensively, reviewing their past medical history and current medication, measuring their BCVA and IOP (Goldmann applanation tonometer, Haag-Streit, Köniz, Switzerland), and performing a slit-lamp examination of the anterior and posterior eye segment and indirect fundoscopy.

**Figure 1:**
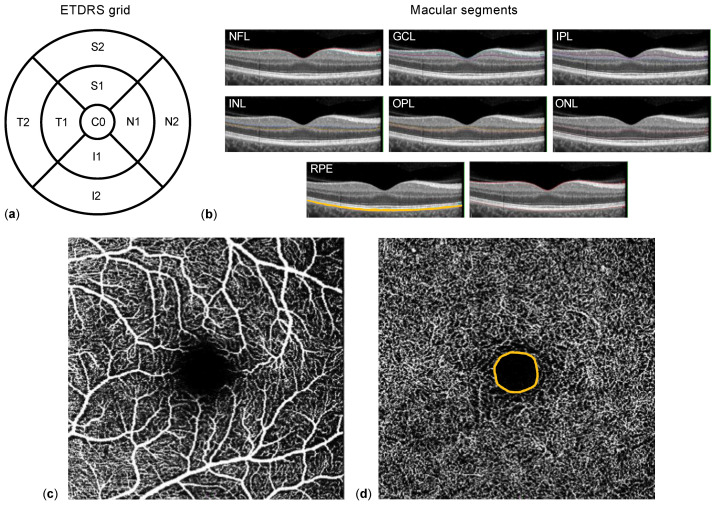
Methodology. (**a**) The Early Treatment Diabetic Retinopathy Study (ETDRS) grid 1, 2.22, 3.45 mm, consisting of nine subfields (C0: center; S1 and S2 superior, N1 and N2 nasal, I1 and I2 inferior, and T1 and T2 temporal). Macular segments (**b**) are separated semi-automatically by the optical coherence tomography (OCT) software: nerve fiber layer (NFL), ganglion cell layer (GCL), inner plexiform layer (IPL), inner nuclear layer (INL), outer plexiform layer (OPL), outer nuclear layer (ONL), and retinal pigment epithelium (RPE). OCT-angiography example of the superficial (**c**) and deep (**d**) capillary plexus of the macula; the orange line indicates the borders of foveal avascular zone.

Macular spectral-domain OCT and OCTA were obtained using a SPECTRALIS^®^ HRA+OCT (Heidelberg Engineering, Heidelberg, Germany). Corneal curvature values (c-curve) were recorded for all patients. Two consecutive examinations of sufficient image quality (quality score ≥ 20) were acquired. Macular OCT scans consisted of 25 single horizontal axial scans centered on the fovea. Individual retinal layer thicknesses were obtained after segmentation of the images using the manufacturer’s software: entire retinal thickness, nerve fiber layer (NFL), ganglion cell layer (GCL), inner plexiform layer (IPL), inner nuclear layer (INL), outer plexiform layer (OPL), outer nuclear layer (ONL), and retinal pigment epithelium (RPE) (Figure 1b). Results of the semi-automated segmentation were inspected separately by two experienced ophthalmologists (Y.C. and M.G.) and, if needed, corrected manually. Thickness results were divided into nine subfields using the Early Treatment Diabetic Retinopathy Study (ETDRS) 1, 2.22, 3.45 mm grid (Figure 1a). Thickness values of each subfield were exported using a software plug-in provided by Heidelberg Engineering (Heidelberg, Germany). Regarding OCTA, the superficial (SCP, extending from the internal limiting membrane to the IPL, Figure 1c) and deep capillary plexus (DCP, extending from the IPL to the OPL, Figure 1d) were acquired automatically. Their respective vessel density (VD) as well as the area of the foveal avascular zone at the DCP level (FAZ, in mm^2^, Figure 1d), were extracted and analyzed by Y.C. and M.G. using ImageJ (Wayne Rasband, version 1.52e) as described by Wang et al. [18].

### 2.3. Statistical Methods

Data were collected in Microsoft Excel (Microsoft, Redmond, WA, USA). Normal distribution was examined using the D’Agostino and Pearson normality test. Mean values of continuous data were compared with the Student’s *t*-test or Mann–Whitney U test, when appropriate. One-way ANOVA was performed to compare multiple subgroups; the Tukey method was chosen for multiple comparison correction in our post hoc analyses. Univariate and multivariate linear regression and other statistical analyses were calculated using Prism 9.3 (GraphPad, La Jolla, CA, USA). In this paper, dichotomous variables are presented as absolute and relative frequencies (*n*, %), categoric variables as median ± interquartile range (IQR), and continuous variables as mean ± standard deviation (SD). Statistical significance was asserted for *p*-values < 0.05.

## 3. Results

### 3.1. Characteristics of the Study Population

A total of 157 healthy participants aged 10 to 89 years (mean 49.8 ± 21.6) were included in this study. Male sex was slightly underrepresented, 41% of participants being men. The mean BCVA was 0.0 ± 0.1 LogMAR. The mean IOP was 15.4 ± 2.9 mm Hg. To compare the evolution of macular segment thickness with age, four age subgroups were formed (15 participants aged 10–19 years, 56 participants aged 20–44 years, 49 participants aged 45–69 years, 37 participants aged 70–90 years). Further epidemiologic characteristics are presented in Table 1.

### 3.2. Changes of Macular Segment Thickness with Age

**Figure 2:**
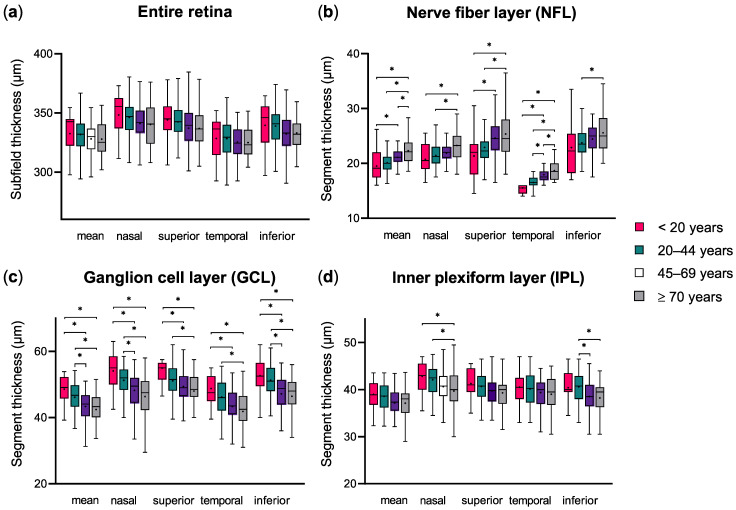
Comparison of macular subfield thickness between age groups. The figure represents the overall, nasal, superior, temporal, and inferior mean macular segment thickness in the four age groups. Statistically different results in the post hoc analysis of variance are represented with an asterisk (*), the mean value is presented with a cross (+).

First, the thickness of macular segments and their ETDRS subfields were examined. To simplify analyses, subfields were averaged into quadrants, e.g., the inner nasal (N1) and outer nasal (N2) averaged into a nasal quadrant. Regarding the entire retinal thickness at the macula, using one-way ANOVA, there was no statistically significant thickness decrease overall. However, the quadrant thickness (nasal, superior, temporal, and inferior) was statistically significantly decreased between the four age subgroups (e.g., superior quadrant: <20 years: 344.1 ± 18.7 µm; 20–44 years: 342.4 ± 16.1 µm; 45–69 years: 337.0 ± 19.0 µm; ≥70 years: 336.8 ± 16.5 µm, Table 2 and Supplementary Table S1). The difference between each subgroup was small, thus the post hoc analysis returned no statistically significant results (Figure 2a). When looking at selected retinal segments, the analysis returned more differentiated results. For the NFL, ANOVA revealed statistical differences between age groups for all four quadrants. Additionally, post hoc analysis showed that NFL thickness increased continuously from the youngest subgroup to the oldest. The difference was statistically significant for many direct subgroup comparisons, particularly between either the <20 years or 20–44 years subgroup and the oldest subgroup (≥70 years) (Figure 2b). A difference between subgroups was also detected for GCL thickness, where the post hoc analysis of ANOVA was statistically significant when comparing the two youngest subgroups with the ≥70 years subgroup and comparing the <20 years with the 45–69 years subgroup (Figure 2c). Comparisons within the IPL thickness data showed statistical differences in the nasal, temporal, and inferior quadrants, but not in the superior. Regarding subgroup comparisons, the nasal IPL thickness in the oldest subgroup differed statistically from the youngest and the 20–44 years subgroups; in the inferior quadrant a statistical difference appeared between the 20–44 years and both the 45–69 years and the oldest subgroups (Figure 2d). Regarding the RPE thickness, the ANOVA identified statistically significant differences between the subgroups in all quadrants; in particular, the 20–44 years subgroup differed from the ≥70 years subgroup in all quadrants and both oldest subgroups were statistically different in the nasal and temporal segments. In general, the direct comparison between the <20 years and the 20–45 years subgroups never reached statistical significance; on the contrary, the oldest subgroup was the one differing most frequently from the others. Analyses of the other macular segments (INL, OPL, ONL) did not show any consistent difference between the age groups and therefore are not presented here.

To further investigate the association between age and macular segment thickness, we performed univariate linear regression analyses. As the ANOVA results suggested, total retinal (macular) thickness as well as thickness of the GCL, IPL, and RPE decreased with age, and the NFL thickness increased with age. The strongest association was seen with the NFL thickness (e.g., temporal quadrant R^2^ = 0.47) and GCL, and was less pronounced within the entire retina, IPL, and RPE data (Table 3). A comparison of the results of right and left eyes is presented in Supplementary Table S2 and of males and females in Supplementary Table S4.

### 3.3. Age-Related Microvascular Changes of the Macula

**Figure 3:**
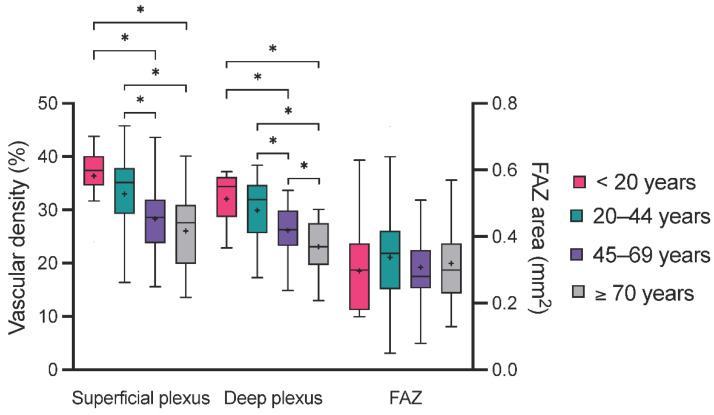
Comparison of OCTA parameters between age groups. The box plots represent the distribution of the vascular density of the superficial and deep plexus as well as of the foveal avascular zone (FAZ) area in all four age groups. Statistically different results in the analysis of variance are represented with an asterisk (*), the mean value is presented with a cross (+).

After observing these age-dependent morphological changes within the macula, we investigated the possible association between age and the macular microvasculature data from OCTA. Using one-way ANOVA, no statistically significant difference of foveal avascular zone area (FAZ) could be detected. However, the VD of the SCP and DCP both decreased between the age subgroups (Table 4). In particular, the post hoc direct comparison revealed statistically significant differences between the <20 years and both the 45–69 years and the ≥70 years subgroups, as well as between the 20–44 years and both older subgroups (Figure 3).

To further quantify the potential association between age and macular VD, univariate linear regression was applied to the OCTA data. No association was detected between age and the FAZ area. However, this analysis revealed a moderate association between age and both the SCP and DCP VD (e.g., DCP: R^2^ = 0.26), where increasing age led to a decrease in VD (Table 5).

### 3.4. Association between Age, Macular Segment Thickness, and OCTA Parameters

Finally, we measured the strength of the association between OCTA parameters and macular segment thickness. The results of multiple linear regression analysis combining the VD of the SCP and DCP to model the mean thickness of the NFL, GCL or IPL returned a strong association, with R^2^ values of 0.21, 0.42, and 0.39, respectively (Table 6). Additionally, using the sum-of-square F test to compare the models, including SVP and DVP alone, against a more complex model including the SVP, DVP, and age returned a P-value of 0.052 and 0.80 for GCL and IPL, respectively. This indicated that the simpler models excluding age provided a better model fit. The association between OCTA parameters (SCP and DCP models) and the whole retinal thickness was also strong (R^2^ = 0.28); however, regarding the deeper segments INL, OPL, and ONL, R^2^ was markedly lower (respectively 0.12, 0.0021, and 0.12). Additionally, a comparison of the linear regression results of right and left eyes is presented in Supplementary Table S3 and of males and females in Supplementary Table S5.

## 4. Discussion

The present study addresses the relationship between age and two clinically central morphologic characteristics of the macula, i.e., macular segment thickness and capillary plexus density measured by OCT(A). The main findings of the study are:

1. Increasing age appears to be associated with a decrease in total macular and inner retinal segment thickness.
2. Superficial and deep plexus vascular density seem to diminish with increasing age.
3. There is a strong association between OCTA parameters and inner macular segment thickness that is independent of age.

Over the course of a lifetime, many changes occur within the eye and the ascending visual pathway. In this study, we investigated the physiological changes in macular morphology and microvasculature in healthy individuals ranging from juvenile (10 years) to older age (89 years).

Through multiple comparisons of homogenous age subgroups and linear regression analysis, the present data showed a clear decrease in the whole macular thickness of approximately 0.13 µm/year. Similarly, other inner retinal segments became significantly thinner with age, most evidently the GCL (0.022 to 0.10 µm/year) and the IPL (0.023 to 0.047 µm/year). This observation was comparable to previous results [27,28] and is explained by the continuous loss of retinal ganglion cells [29,30], of which the cell body and dendrites are located in the GCL and IPL, respectively [31]. Notably, these age-related changes are visible in the perifoveal and parafoveal region but not in the foveal C0 subfield of the ETDRS grid, which was also determined by other groups [30,32]. A similar thickness decreasing trend with age was also observed within the RPE (0.025 to 0.037 µm/year), which was in line with previous histological [29] and OCT studies [28,33]. In the present cohort, the NFL thickness was also influenced by age. Unexpectedly, however, NFL thickness increased with age. In the past, this counterintuitive observation was also made by Demirkaya et al. [30]. Similar results were also presented by Xu et al. [33], even though the authors did not comment on them. The NFL was also reported to remain constant over time [34], or, using older Stratus OCT technology, to decrease with age [6]. Currently, there is no general hypothesis or understanding regarding why NFL thickness should increase with age, and this observation could be caused by measurement imprecision. Regarding other (deeper) macular segments, the present analyses did not provide generalizable age-related changes. The current literature is also divided on this: Wang et al. reported statistically significant age-related changes in the INL and ONL in the perifoveal and parafoveal regions, but Demirkaya et al. did not observe any correlation with age for the parafoveal INL and ONL. One major factor for this disparity in results could be the technical segmentation differences in instruments of various manufacturers. Finally, the age range of the included participants could also influence the observations, particularly when individuals in an extreme age range such as <18 years and >80 years are included, particularly since in our study the subgroup of individuals ≥70 years of age was the one that differed most frequently from the other subgroups.

Regarding OCTA parameters, the presented one-way ANOVA showed a continuous decrease in the SCP and DCP VD from younger to older age subgroups. This observation translated into a marked association of these parameters with age when applying univariate linear regression. The present results of a wide age range (10–79 years) were consistent with previous reports from adult-only cohorts [18,19] and studies including data from children [35,36]. An additional decrease in SCP and DCP VD was also observed in other degenerative and vascular eye conditions such as glaucoma [11,23] and anterior ischemic optic neuropathy [24]. Thus, the physiological decay of the macular capillary VD over a lifetime must be acknowledged when interpreting OCTA measurements. Concerning the FAZ, in the present study, its area remained constant over time. This was consistent with several previous studies [11,18,37], even though a few groups reported a slight increase in FAZ area with increasing age [35,38]. The stability of the FAZ area can be explained by the lack of retinal layers, except for photoreceptors in the foveola [34], the anatomical structure corresponding to the FAZ and C0 subfield of the ETDRS grid. This is particularly interesting since the FAZ area was shown to increase in the context of macular ischemia, such as in diabetic [20,21] and radiation retinopathy [39], and following central vein occlusion [20]. Thus, at any age, small changes in the FAZ area over time can be indicative of a macular microangiopathy.

Finally, another important aspect is the correlation between morphological changes detected through macular segmentation with SD-OCT and microvascular alterations visible with OCTA. The present analyses showed an association between the whole retinal thickness as well as the thickness of the NFL, GCL and IPL segments with the VD of the SCP and DCP. This association was the most constant for the GCL and IPL. This correlation between inner macular layers and OCTA parameters was also observed in studies with open-angle [11,23] and normal tension glaucoma [40] patients and can be explained histologically as the SCP and DCP are located between the GCL and IPL. It is possible that this association was only mediated by age, but the present analysis does not support this hypothesis as adjusting our regression analysis for the individuals’ ages by adding this variable to the regression model did not improve the strength of association (R^2^).

The present study has several limitations that impede the generalizability of its results. First, the retrospective study design cannot provide the same level of validity or reliability as a longitudinal prospective study. Additionally, OCT(A) examinations were performed at different times of the day, and it was not possible to control or adjust for a potential physiological diurnal variation in OCT(A) parameters [41]. In addition, the individuals included were all healthy, with only slight refraction errors, which does not reflect the variety of the general population. The comparability of this study with others is made difficult by the choice of a 3 × 3 mm scan size for OCTA measurements; in general, the comparability of OCTA analyses between various instruments is difficult because of differing segmentation algorithms included in the manufacturers’ software. Finally, the age groups chosen in this study are unusual as adults are often separated into 10- or 20-year subgroups, often leading to an “old” age group containing individuals ≥60 years old; here, individuals of 45–69 years are regrouped and the “old” group starts at 70 years of age, which seemed better suited, as macular segment thickness was quite stable in individuals between 20 and 70 years of age—the most visible changes occurring not from 60 years on, but from 70 years of age.

## 5. Conclusions

In conclusion, the present study supports and extends knowledge from previous studies describing the decrease in selected inner retinal segments and in macular vascular density from childhood to old age. The present results also highlight the importance of the association between inner retinal segment thickness data and OCTA parameters, which seems age-independent. This must be accounted for when interpreting measurements in healthy individuals but also in the context of ocular pathologies. Finally, this study provides additional macular segmentation and OCTA data of healthy children and adults, yet normative databases for clinical application are still missing.

## Figures and Tables

**Table 1:** Epidemiologic and ophthalmologic characteristics of the whole study population and of separated age subgroups.

	Whole Population	Subgroups			
		<20 Years	20–44 Years	45–69 Years	≥70 Years
Patients (n)	157	15	56	49	37
Sex [male: female% (n)]	41.4:58.6% (65:92)	67:33% (10:5)	36:64% (20:36)	51:49% (25:24)	41:59% (15:22)
Eye [right: left% (n)]	48.4:51.6% (76:81)	47:53% (7:8)	41:59% (23:33)	45:55% (27:22)	51:49% (19:18)
Age [mean ± SD (y)]	49.8 ± 21.6	14.5 ± 3.0	32.0 ± 6.8	58.9 ± 6.3	76.8 ± 5.0
BCVA [mean ± SD (LogMar)]	0.0 ± 0.06	0.0 ± 0.02	0.0 ± 0.11	0.1 ± 0.08	0.1 ± 0.11
IOP [mean ± SD (mm Hg)]	15.4 ± 2.9	15.4 ± 2.8	15.9 ± 2.6	15.0 ± 2.8	14.5 ± 3.4

Abbreviations: BCVA, best corrected visual acuity; IOP, intraocular pressure; y, years; SD, standard deviation; IQR, interquartile range.

**Table 2:** Macular segment thickness differs between age groups. The table presents the mean thickness of selected macular segment quadrants and the corresponding results of one-way ANOVA.

Macular Segment	Mean Thickness (µm ± SD)				ANOVA Summary		
	<20 Years	20–44 Years	45–69 Years	≥70 Years	F	R^2^	*p*-Value
Retina							
Nasal	348.3 ± 18.1	346.3 ± 15.6	341.0 ± 16.1	340.3 ± 18.4	45.9	0.36	**<0.0001**
Superior	344.1 ± 18.7	342.4 ± 16.1	337.0 ± 19.0	336.8 ± 16.5	45.8	0.36	**<0.0001**
Temporal	328.5 ± 19.0	328.3 ± 15.9	325.3 ± 16.2	324.9 ± 13.1	39.4	0.32	**<0.0001**
Inferior	339.5 ± 21.2	338.9 ± 15.8	332.3 ± 16.2	332.9 ± 14.0	48.1	0.37	**<0.0001**
Nerve fiber layer (NFL)							
Nasal	20.7 ± 2.7	21.2 ± 2.4	22.1 ± 2.4	23.6 ± 3.8	5.6	0.098	**0.0011**
Superior	21.3 ± 4.2	22.9 ± 2.8	24.6 ± 3.7	25.5 ± 5.0	6.9	0.12	**0.0002**
Temporal	15.5 ± 1.1	16.5 ± 1.4	17.9 ± 1.1	18.8 ± 2.1	3.6	0.39	**<0.0001**
Inferior	22.9 ± 4.8	23.9 ± 3.0	24.4 ± 3.5	25.6 ± 3.5	3.3	0.060	**0.022**
Ganglion cell layer (GCL)							
Nasal	54.1 ± 5.4	51.3 ± 4.7	48.4 ± 7.8	46.7 ± 7.1	9.8	0.16	**<0.0001**
Superior	55.0 ± 5.5	51.1 ± 5.0	47.9 ± 8.1	48.2 ± 5.9	6.7	0.12	**0.0003**
Temporal	48.8 ± 4.6	46.2 ± 5.3	43.5 ± 7.2	42.3 ± 6.9	7.7	0.13	**<0.0001**
Inferior	52.6 ± 6.2	51.3 ± 4.8	47.9 ± 7.9	46.8 ± 6.7	9.0	0.15	**<0.0001**
Inner plexiform layer (IPL)							
Nasal	42.8 ± 3.1	42.1 ± 3.2	40.3 ± 4.2	40.0 ± 4.6	4.5	0.080	**0.0046**
Superior	41.3 ± 2.9	40.7 ± 3.1	38.9 ± 4.9	39.4 ± 3.9	2.0	0.038	0.11
Temporal	40.5 ± 3.4	40.1 ± 3.3	39.2 ± 4.6	39.1 ± 3.8	1.3	0.024	**0.024**
Inferior	40.4 ± 3.8	40.6 ± 3.0	38.5 ± 3.9	38.4 ± 3.7	5.0	0.087	**0.0024**
Retinal pigment epithelium (RPE)							
Nasal	83.0 ± 2.0	83.4 ± 2.3	83.0 ± 2.6	81.4 ± 3.1	6.0	0.11	**0.0007**
Superior	81.6 ± 2.4	82.4 ± 2.3	82.0 ± 2.8	80.7 ± 2.7	4.0	0.073	**0.0092**
Temporal	81.5 ± 1.9	82.1 ± 2.1	82.0 ± 2.4	80.6 ± 2.5	4.9	0.088	**0.0027**
Inferior	81.0 ± 2.1	81.4 ± 2.1	80.9 ± 2.4	79.7 ± 2.5	5.1	0.091	**0.0023**

*p*-values of ANOVA are bold and underlined when statistically significant (*p* < 0.05). Abbreviation: ANOVA, analysis of variance; SD, standard deviation.

**Table 3:** Macular segment thickness is associated with age. The table displays the results of univariate linear regression between age groups and the mean thickness of selected macular segment quadrants.

	Parameter Estimate	95% CI	R^2^	*p*-Value
Retina				
Nasal	−0.13	−0.25 to −0.010	0.029	**0.034**
Superior	−0.12	−0.25 to 0.0051	0.023	0.060
Temporal	−0.064	−0.18 to 0.050	0.0079	0.27
Inferior	−0.13	−0.25 to −0.016	0.031	**0.026**
Nerve fiber layer (NFL)				
Nasal	0.035	0.018 to 0.053	0.091	**0.0001**
Superior	0.056	0.031 to 0.082	0.11	**<0.0001**
Temporal	0.052	0.043 to 0.060	0.47	**<0.0001**
Inferior	0.042	0.017 to 0.067	0.068	**0.0011**
Ganglion cell layer (GCL)				
Nasal	−0.022	−0.15 to −0.063	0.13	**<0.0001**
Superior	−0.082	−0.12 to −0.040	0.089	**0.0002**
Temporal	−0.096	−0.14 to −0.054	0.12	**<0.0001**
Inferior	−0.10	−0.14 to −0.059	0.13	**<0.0001**
Inner plexiform layer (IPL)				
Nasal	−0.047	−0.075 to −0.018	0.064	**0.0014**
Superior	−0.030	−0.058 to −0.0029	0.030	**0.031**
Temporal	−0.023	−0.047 to 0.0024	0.021	0.076
Inferior	−0.043	−0.068 to −0.018	0.069	**0.0009**
Retinal pigment epithelium (RPE)				
Nasal	−0.037	−0.057 to −0.018	0.083	**0.0003**
Superior	−0.026	−0.045 to −0.0073	0.047	**0.0068**
Temporal	−0.025	−0.042 to −0.0082	0.053	**0.0038**
Inferior	−0.032	−0.049 to −0.015	0.081	**0.0003**

Abbreviations: 95% CI, 95% confidence interval of the parameter estimate; R^2^, Tjur’s pseudo R^2^. *p*-values are bold and underlined when statistically significant (*p* < 0.05).

**Table 4:** Macular capillary plexus density differs between age groups. The table presents the mean value of the superficial and deep plexus capillary density and of the foveal avascular zone (FAZ) area in all age groups and corresponding results of one-way ANOVA.

	Subgroups				ANOVA Summary		
	<20 Years	20–44 Years	45–69 Years	≥70 Years	F	R^2^	*p*-Value
FAZ area (mm^2^)	0.30 ± 0.13	0.34 ± 0.14	0.31 ± 0.12	0.32 ± 0.11	0.71	0.014	0.55
SCP vascular density (%)	36.4 ± 4.8	33.0 ± 6.6	28.4 ± 5.9	26.1 ± 6.9	15.2	0.23	**<0.0001**
DCP vascular density (%)	32.1 ± 4.8	30.0 ± 6.0	26.2 ± 4.6	23.1 ± 4.8	18.0	0.26	**<0.0001**

Abbreviation: FAZ, foveal avascular zone; 95% CI, 95% confidence interval of the mean difference; SCP, superficial capillary plexus; DCP, deep capillary plexus. *p*-values are bold and underlined when statistically significant (*p* < 0.05).

**Table 5:** Quantification of the association between OCTA parameters and age. The table displays the results of univariate linear regression between the age of individuals and optical coherence tomography-angiography (OCTA) parameters.

	Parameter Estimate	95% CI	R^2^	*p*-Value
FAZ area	−0.00013	−0.0010 to 0.00078	0.00053	0.77
SCP vascular density	−0.15	−0.20 to −0.11	0.22	**<0.0001**
DCP vascular density	−0.14	−0.18 to −0.10	0.26	**<0.0001**

Abbreviations: FAZ, foveal avascular zone; 95% CI, 95% confidence interval of the parameter estimate; R^2^, Tjur’s pseudo R^2^; SCP, superficial capillary plexus; DCP, deep capillary plexus. *p*-values are bold and underlined when statistically significant (*p* < 0.05).

**Table 6:** Association between OCTA parameters and selected inner retinal layers. The table displays the results of multivariate linear regression between the vascular density of the macular superficial and deep capillary plexus and the thickness of the whole retina, NFL, GCL, IPL, and RPE.

	Parameter Estimate	95% CI	*p*-Value	R^2^
Retina				
Superficial plexus	1.5	0.98 to 2.0	**<0.0001**	0.28
Deep plexus	−0.55	−1.2 to 0.052	0.073	
NFL				
Superficial plexus	0.19	0.11 to 0.26	**<0.0001**	0.21
Deep plexus	−0.29	−0.38 to −0.20	**<0.0001**	
GCL				
Superficial plexus	0.80	0.63 to 0.97	**<0.0001**	0.42
Deep plexus	−0.49	−0.69 to −0.29	**<0.0001**	
IPL				
Superficial plexus	0.44	0.34 to 0.55	**<0.0001**	0.39
Deep plexus	−0.25	−0.37 to −0.13	**<0.0001**	
RPE				
Superficial plexus	0.00044	−0.095 to 0.096	0.99	0.0029
Deep plexus	0.022	−0.092 to 0.14	0.71	

Abbreviations: NFL, nerve fiber layer; GCL, ganglion cell layer; IPL, inner plexiform layer; RPE, retinal pigment epithelium; 95% CI, 95% confidence interval of the parameter estimate. *p*-values are bold and underlined when statistically significant (*p* < 0.05).

## Data Availability

The data supporting the reported results can be provided by the corresponding author on reasonable request.

## Supplementary Materials

The following supporting information can be downloaded at: https://www.mdpi.com/article/10.3390/life12040568/s1, Table S1: Macular segment thickness (µm) and OCTA parameters differ between age groups. Table S2: Mean, minimal, and maximal values for macular segment thickness (µm) and OCTA parameters, comparison of right (OD) and left eyes (OS). Table S3: Association between OCTA parameters and selected inner retinal layers, comparison of right and left eyes. Table S4: Mean, minimal, and maximal values for macular segment thickness (µm) and OCTA parameters, comparison of male and female individuals. Table S5: Association between OCTA parameters and selected inner retinal layers, comparison of males and females.
